# Lenvatinib in combination with radiotherapy versus lenvatinib with transarterial chemoembolization for advanced hepatocellular carcinoma

**DOI:** 10.1186/s12885-025-14931-1

**Published:** 2025-09-30

**Authors:** Wen-Yu Chuang, Po-Chien Shen, Sung-Hua Chiu, Wen-Yen Huang, Wei-Chou Chang, Chun-Shu Lin, Cheng-Hsiang Lo

**Affiliations:** 1 Department of Radiation Oncology, Tri‑Service General Hospital, National Defense Medical University, No. 325, Sec 2, Cheng-Gong Rd, Neihu, Taipei, 114 Taiwan; 2 Department of Radiology, Tri-Service General Hospital, National Defense Medical University, Taipei, Taiwan

**Keywords:** Combined modality therapy, Hepatocellular carcinoma, Lenvatinib, Radiotherapy, Transarterial chemoembolization

## Abstract

**Background:**

This study aimed to evaluate the efficacy of lenvatinib combined with either radiotherapy (RT) or transarterial chemoembolization (TACE) in patients with advanced hepatocellular carcinoma (HCC).

**Methods:**

Conducted between December 2018 and January 2022, this retrospective study included 32 patients with advanced HCC from a single institution. The patients were divided into two treatment groups: RT plus lenvatinib (*n* = 17) and TACE plus lenvatinib (*n* = 15). The primary outcomes assessed were overall survival (OS) and infield control (IFC). Treatment modalities, patient demographics, disease characteristics, and therapeutic responses were analyzed using the Kaplan–Meier method and Cox regression models to identify predictors of OS and IFC. To address baseline imbalances and competing risks, inverse-probability-of-treatment weighting (IPTW) and Fine–Gray analyses were applied to better estimate IFC outcomes.

**Results:**

With a median follow-up of 10.2 months, no significant difference in OS was observed between the RT and TACE groups. However, the Kaplan–Meier analysis indicated significantly longer IFC durations in the RT group (*p* = 0.010), with a 1-year IFC rate of 74.7% compared to 13.2% in the TACE group. Multivariable analysis further demonstrated that the RT group was associated with better IFC outcomes (*p* = 0.023). After IPTW adjustment, the RT group retained a significant IFC benefit (*p* = 0.020). At 12 months, the cumulative infield failure rate was 61.0% in the TACE group versus 14.9% in the RT group (*p* = 0.015). Alpha-fetoprotein levels significantly declined within 3 months after RT (*p* = 0.002) but not after TACE (*p* = 0.510). A ≥ 2-point deterioration in the Child–Pugh score was observed in 5.9% of the RT group when compared to 26.7% of the TACE group (*p* = 0.161).

**Conclusion:**

These findings suggest that RT combined with lenvatinib may offer advantages in local tumor control and potentially liver function preservation, providing a promising alternative for patients with advanced HCC.

**Supplementary Information:**

The online version contains supplementary material available at 10.1186/s12885-025-14931-1.

## Background

Primary Liver cancer poses a significant global health challenge. It ranks as the sixth most commonly diagnosed cancer worldwide and has the fourth leading cause of cancer-related deaths. The number of annual Liver cancer cases is projected to exceed one million by 2025 [1], with annual deaths expected to Surpass one million by 2030 [2]. Hepatocellular carcinoma (HCC) constitutes at least 90% of all primary liver cancer cases. The primary risk factors for HCC include chronic hepatitis B or C viral infections, excessive alcohol intake, and non-alcoholic fatty liver disease (NAFLD) [3].

Various local treatments are currently available for HCC, including surgical resection, transplantation, ablation, embolization, and radiotherapy (RT). Approximately one-third of treatment-naïve patients with HCC are eligible for surgery or transplantation. However, most patients rely on alternative treatment approaches. Several studies have established the efficacy of RT, either as a stand-alone or adjunctive treatment for embolization or radiofrequency ablation, showing favorable outcomes in terms of survival and local tumor control [4–6]. Nonetheless, significant challenges remain in treating HCC cases with adverse features of diffuse tumors, macrovascular invasion, or extrahepatic metastasis, where current therapeutic strategies are often insufficient.

Lenvatinib, an oral multi-kinase receptor targeting inhibitor, has been widely used in unresectable HCC, as evidenced by the phase III REFLECT trial [7, 8]. In this trial, lenvatinib achieved a median overall survival (OS) of 13.6 months, marginally outperforming that of 12.3 months with sorafenib, thus fulfilling the non-inferiority criteria. Moreover, the lenvatinib cohort showed significantly improved median time to progression, objective response rate, and progression-free survival. Consequently, lenvatinib has been approved as a first-line treatment for unresectable HCC in various regions, including the United States [9], European Union [10], Japan [11], and China [12], marking a significant advancement in therapeutic approaches. In an Asian cohort of patients with unresectable HCC, lenvatinib exhibits efficacy and safety comparable to the combination of atezolizumab plus bevacizumab when used as first-line systemic therapy [13]. Notably, lenvatinib significantly improves survival when compared to atezolizumab plus bevacizumab in patients with HCC resulting from NAFLD/nonalcoholic steatohepatitis [14].

Studies indicate that inhibitors of tumor angiogenesis may augment the radiosensitivity of hepatocellular tumors, suggesting a potential synergistic effect when combined with RT [15, 16]. While the combination of transarterial chemoembolization (TACE) and lenvatinib yielded promising outcomes in the LAUNCH trial [17], direct comparisons between RT and TACE, in conjunction with lenvatinib, remain scarce. This study aimed to fill this research gap by comparing the efficacy of the combination therapies of lenvatinib with either RT or TACE in patients with advanced HCC.

## Methods

### Patients

This retrospective study utilized data of patients with HCC recorded in the cancer registries of the Tri-Service General Hospital in Taiwan. Medical records of patients treated with lenvatinib in combination with either RT or TACE between December 2018 and January 2022 were reviewed. Treatment decisions were typically made by a multidisciplinary team, incorporating factors such as portal vein tumor thrombosis (PVTT), tumor accessibility, liver function, and prior treatment response. Patients were informed of the respective advantages and limitations, and the final decision was made in accordance with patient preference and clinical feasibility. The inclusion criteria were as follows: (1) confirmed HCC diagnosis based on radiological or pathological criteria; (2) administration of lenvatinib with either RT or TACE within a two-month window; (3) absence of scheduled liver transplantation or surgical intervention plan; and (4) absence of any concurrent immunotherapy medication. The patient demographics and clinical details are shown in Table 1. Institutional review board approval (TSGHIRB No.: B202405172) was obtained for this study, and the requirement for informed consent was waived owing to its retrospective nature.

**Table 1:** Patient, tumor, and treatment characteristics

Characteristics	RT group (*n* = 17)	TACE group (*n* = 15)	*p*
Age, median (range), y	64 (28–83)	61 (45–78)	0.748
Gender			0.678
Female	3 (17.6%)	4 (26.7%)	
Male	14 (82.4%)	11(73.3%)	
ECOG			0.291
0	9 (52.9%)	11 (73.3%)	
≥1	8 (47.1%)	4 (26.7%)	
Etiology			0.099
HBV	9 (52.9%)	13 (86.7%)	
HCV	2 (11.8%)	0 (0%)	
Others	6 (35.3%)	2(13.3%)	
BCLC stage			0.144
B	4 (23.5%)	8 (53.3%)	
C	13 (76.5%)	7 (46.7%)	
Index tumor size, median (range), cm	4.6 (1.4–11)	3.6 (1.3–18.1)	0.720
Tumor number			1.000
Solitary	4 (23.5%)	4 (26.7%)	
Multiple	13 (76.5%)	11 (73.3%)	
Tumor location			0.308
Unilateral	8 (47.1%)	10 (66.7%)	
Bilateral	9 (52.9%)	5 (33.3%)	
Extrahepatic metastasis	4 (23.5%)	2 (13.3%)	0.659
PVTT	10 (58.8%)	4 (26.7%)	0.087
ALBI grade			0.712
1	5 (29.4%)	6 (40%)	
2–3	12 (70.6%)	9 (60%)	
AFP ≥ 400 ng/ml	5 (29.4%)	2 (13.3%)	0.402
Recurrent disease	15 (88.2%)	10 (66.6%)	0.209
Treatment sequence			
LT-first^a^	7 (41.2%)	3 (20.0%)	0.265
LEN-first^b^	10 (58.8%)	12 (80.0%)	

*Abbreviations*: *AFP* Alpha-fetoprotein, *ALBI* Albumin-bilirubin, *BCLC* Barcelona Clinic Liver Cancer, *ECOG* Eastern Cooperative Oncology Group, *HBV* Hepatitis B virus, *HCV* Hepatitis C virus, *LEN* Lenvatinib, *LT* Local therapy, *PVTT* Portal vein tumor thrombosis, *RT* Radiotherapy, *TACE* Transarterial chemoembolization

^a^Local therapy (RT or TACE) followed by subsequent lenvatinib

^b^Lenvatinib was initiated prior to and maintained during RT/TACE

### RT technique

For computed tomography (CT) simulation, the patients were positioned supine, immobilized with a vacuum cushion, and scanned with their arms raised overhead. Motion management utilizing breath-hold techniques with an active breathing control (ABC) system was encouraged, if tolerable. Four-dimensional CT was used for patients who were unable to hold their breath effectively. The RT regimen of either conventional fractionated radiotherapy (CFRT) or stereotactic body radiation therapy (SBRT) was determined by a radiation oncologist based on liver function, tumor size, location, and other patient-specific factors. Treatment planning was conducted using the Philips Pinnacle v9.2 planning system. Radiation therapy was administered using an Elekta Synergy or Versa HD linear accelerator (Elekta AB; Stockholm, Sweden).

In patients using an ABC system, the gross tumor volume (GTV) was determined as the tumor visible on radiographic images, identified through contrast enhancement on CT or magnetic resonance imaging (MRI). No additional clinical target volume (CTV) margin was applied to the GTV. For the planning target volume (PTV), an additional margin of 8–10 mm radially and 5–10 mm craniocaudally was added to the GTV.

For patients who underwent four-dimensional CT during simulation, the internal target volume was estimated based on target motion and then expanded to the PTV in the same manner as patients treated using the ABC technique [18].

### DEB-TACE procedure

In this study, the TACE procedure was implemented following an “on-demand” approach, tailored according to the institution’s guidelines [19], based on tumor burden, liver function, and patient tolerance. DEB-TACE was performed under the guidance of experienced interventional radiologists using Siemens Axiom Artis equipment. The procedure involved several steps: digital subtraction angiography to map vessels, diagnostic visceral angiography, cone-beam CT during hepatic arteriography, and selective catheterization of arteries feeding the tumor. For embolization, two types of beads were used: DC Beads (70–150 μm, BTG) and Hepasphere beads (30–60 μm, Merit Medical), both loaded with doxorubicin, complemented by Gelfoam sponges as adjuvant embolizers. The goal was to achieve diminished residual arterial flow and tumor blush, evaluated using the subjective angiographic chemoembolization endpoint scale. Post-procedure, non-enhanced cone-beam CT was used to assess the saturation of contrast material across the tumor.

### Lenvatinib

The initial dose of lenvatinib was determined based on body weight; patients weighing less than 60 kg started at 8 mg daily, whereas those weighing 60 kg or above began at 12 mg daily. Lenvatinib is administered orally once daily until disease progression or unacceptable toxicity occurs. In the event of severe (grade 3/4) toxicities, such as hypertension, cardiac dysfunction, hepatotoxicity, or renal failure, the dosage may be reduced, or the treatment may be discontinued by the primary physician. Lenvatinib treatment can commence before, during, or after the course of RT/TACE, provided the interval between these therapies does not exceed 2 months.

### Assessment and follow-up

All patients underwent abdominal CT or MRI at 1-, 3-, and 6-months following completion of RT or TACE, followed by imaging every 3–4 months thereafter. Clinical evaluations and blood tests were conducted on a monthly basis. Serum alpha-fetoprotein (AFP) levels were assessed at baseline, within the first 3 months following the completion of local therapy (LT), and Subsequently at 3-month intervals during follow-up. Patients who experienced disease progression were offered salvage or palliative treatments. Treatment response was assessed using the modified Response Evaluation Criteria for Solid Tumors (mRECIST) [20]. The National Cancer Institute Common Terminology Criteria for Adverse Events (CTCAE, version 5.0) was used to evaluate adverse events [21].

Infield control (IFC) was evaluated based on the index lesion response for each patient, defined as the duration from the day of the initial RT or TACE session until the occurrence of progressive disease or new enhancement within or around the treatment field—defined for RT as the PTV plus an additional 1.5 cm [22], and for TACE as the embolization zone—or until the last follow-up. The index lesion was defined as the largest tumor treated with RT or TACE. OS was calculated from the date of the first RT or TACE session until death from any cause or the last follow-up. Data were censored at the time of liver transplantation in the OS and IFC analysis.

### Statistical analysis

The Mann–Whitney U and Fisher’s exact test were used to compare continuous and categorical variables, respectively, between the two study groups. The difference in AFP values pre- and post-LT was evaluated using the Wilcoxon signed-rank test. Kaplan–Meier (KM) analysis was used to calculate the OS and IFC rates, and differences were evaluated using the log-rank test. Univariable Cox proportional hazards models were used to identify prognostic factors for IFC and OS across the cohort. Factors with *p* < 0.1 were included in the multivariable analyses using a backward selection method to avoid missing important confounders. All statistical tests were two-sided, and a *p* value < 0.05 was considered statistically significant. All analyses described above were performed using SPSS version 22 software (SPSS Inc., Chicago, IL, USA).

To address baseline imbalances between the two treatment groups, inverse-probability-of-treatment weighting (IPTW) was applied. The covariates used for IPTW adjustment included variables with a *p* value < 0.1 as well as clinically relevant factors with notable differences in patient characteristics. Additionally, competing risk analysis using the cumulative incidence function and Fine–Gray test was performed to more accurately evaluate IFC outcomes. All analyses described above were conducted using R version 4.2.0 (The R Foundation for Statistical Computing, Vienna, Austria).

## Results

### Patient characteristics

This study included 32 patients, of whom 17 (53.1%) received a combination of RT and lenvatinib (Table 1). The analysis revealed that the two groups did not display significant differences in demographics, Liver disease etiology, tumor characteristics, or recurrent disease. In the RT group, 7 patients underwent SBRT and 10 underwent CFRT. CFRT regimens comprise 15–25 sessions of conventional fractionation, delivering a total dose of 40–68 Gray (Gy), with a median dose of 45 Gy. SBRT involves 5–6 fractions, delivering a total dose of 35–50 Gy (median, 44 Gy).

Two treatment sequences were defined in our cohort: (1) LEN-first, in which lenvatinib was initiated prior to and maintained during RT or TACE (*n* = 22); and (2) LT-first, in which RT or TACE was administered first, followed by the subsequent initiation of lenvatinib (*n* = 10). These two groups were used in subsequent analyses to assess whether the timing of lenvatinib administration relative to LT influenced treatment outcomes. Further details on dose constraints can be found in our previous study [18].

Prior to IPTW, several covariates exhibited substantial imbalance between the two groups. For example, HCC etiology had a standardized mean difference (SMD) of 0.666, indicating a notable baseline discrepancy. After IPTW adjustment, covariate balance was markedly improved, with most SMDs reduced to below 0.1, Suggesting effective mitigation of confounding. The effective sample sizes were 31 in the RT group and 29 in the TACE group. A summary table of covariate balance before and after IPTW is provided in Supplementary Table S1.

### Survival

**Fig. 1:**
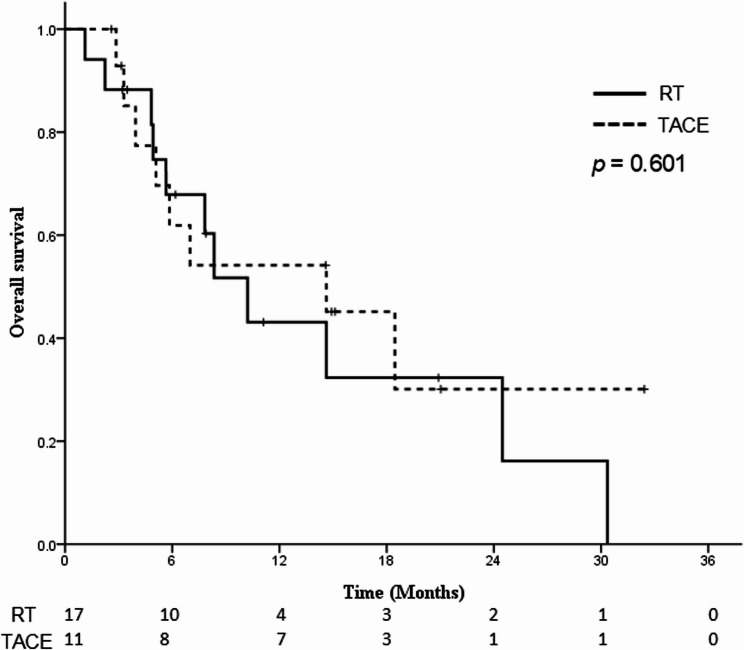
Kaplan-Meier curves of OS for patients in the RT and TACE groups

The median follow-up time was 10.2 months. The KM analysis showed that the RT group had a median OS of 10.2 months (95% confidence interval [CI]: 6.5–14 months), with 1-year OS rates of 43.1%. In comparison, the TACE group had a median OS of 14.6 months (95% CI: 3.6–25.6 months), with 1-year OS rates of 54.2%. However, these differences were not statistically significant (*p* = 0.601) (Fig. 1). Multivariable analysis (Table 2; Supplementary Figure S1) revealed that the presence of extrahepatic metastasis (hazard ratio [HR], 4.87; 95% CI, 1.42–16.66; *p* = 0.012) was an independent predictor of inferior OS, whereas an ALBI grade ≥ 2 predicted better OS (HR, 0.19; 95% CI, 0.06–0.66; *p* = 0.009). Eastern Cooperative Oncology Group (ECOG) performance status $\:\ge\:$1 demonstrated a trend toward for inferior OS (*p* = 0.054). After IPTW, no statistically significant difference in OS was observed between the RT and TACE groups (*p* = 1.000), suggesting that long-term survival outcomes were statistically comparable following adjustment for baseline confounders (Supplementary Figure S2).

**Table 2:** Univariable and multivariable analysis of OS and IFC

Variable	OS				IFC			
	Univariable		Multivariable		Univariable		Multivariable	
	HR (95% CI)	*p*	HR (95% CI)	*p*	HR (95% CI)	*p*	HR (95% CI)	*p*
RT vs. TACE	1.27 (0.51–3.19)	0.604			0.16 (0.03–0.78)	0.023	0.16 (0.03–0.78)	0.023
Age$\:\:\ge\:$60, y	3.22 (1.02–10.21)	0.047			1.41 (0.40–5.04)	0.597		
Female vs. male	1.45 (0.47–4.52)	0.519			1.63 (0.32–8.29)	0.555		
ECOG$\:\ge\:$1	2.32 (0.91–5.91)	0.078	2.75 (0.98–7.69)	0.054	0.64 (0.16–2.47)	0.514		
Etiology								
Viral vs. non-viral	0.95 (0.28–2.81)	0.927			0.03 (0.00–13.20)	0.260		
Tumor size$\:\ge\:$5 cm	0.64 (0.24–1.74)	0.382			3.59 (0.91–14.20)	0.068		
Tumor number								
Multiple vs. solitary	0.95 (0.33–2.74)	0.923			1.45 (0.30–6.90)	0.644		
Tumor location								
Bilateral vs. unilateral	0.43 (0.15–1.24)	0.120			0.83 (0.22–3.09)	0.780		
Extrahepatic metastasis	3.59 (1.23–10.44)	0.019	4.87 (1.42–16.66)	0.012	0.04 (0.00–73.36)	0.398		
PVTT	2.29 (0.79–6.62)	0.128			1.56 (0.36–6.81)	0.553		
ALBI grade$\:\ge\:$2	0.43 (0.15–1.17)	0.099	0.19 (0.06–0.66)	0.009	0.48 (0.11–2.07)	0.325		
AFP$\:\:\ge\:$400 ng/ml	1.55 (0.55–4.43)	0.409			1.09 (0.21–5.35)	0.935		
Recurrent disease	1.05 (0.34–3.24)	0.932			0.92 (0.19–4.37)	0.916		
Treatment sequence								
LT-first^a^ vs. LEN-first^b^	0.69 (0.25–1.93)	0.486			1.26 (0.35–4.49)	0.720		

*Abbreviations*: *AFP* Alpha-fetoprotein, *ALBI* Albumin-bilirubin, *ECOG* Eastern Cooperative Oncology Group, *HBV* Hepatitis B virus, *HCV* Hepatitis C virus, *IFC* Infield control, *LEN* Lenvatinib, *LT* Local therapy, *OS* Overall survival, *PVTT* Portal vein tumor thrombosis, *RT* Radiotherapy, *TACE* Transarterial chemoembolization

^a^Local therapy (RT or TACE) followed by subsequent lenvatinib

^b^Lenvatinib was initiated prior to and maintained during RT/TACE

### Infield control

**Fig. 2:**
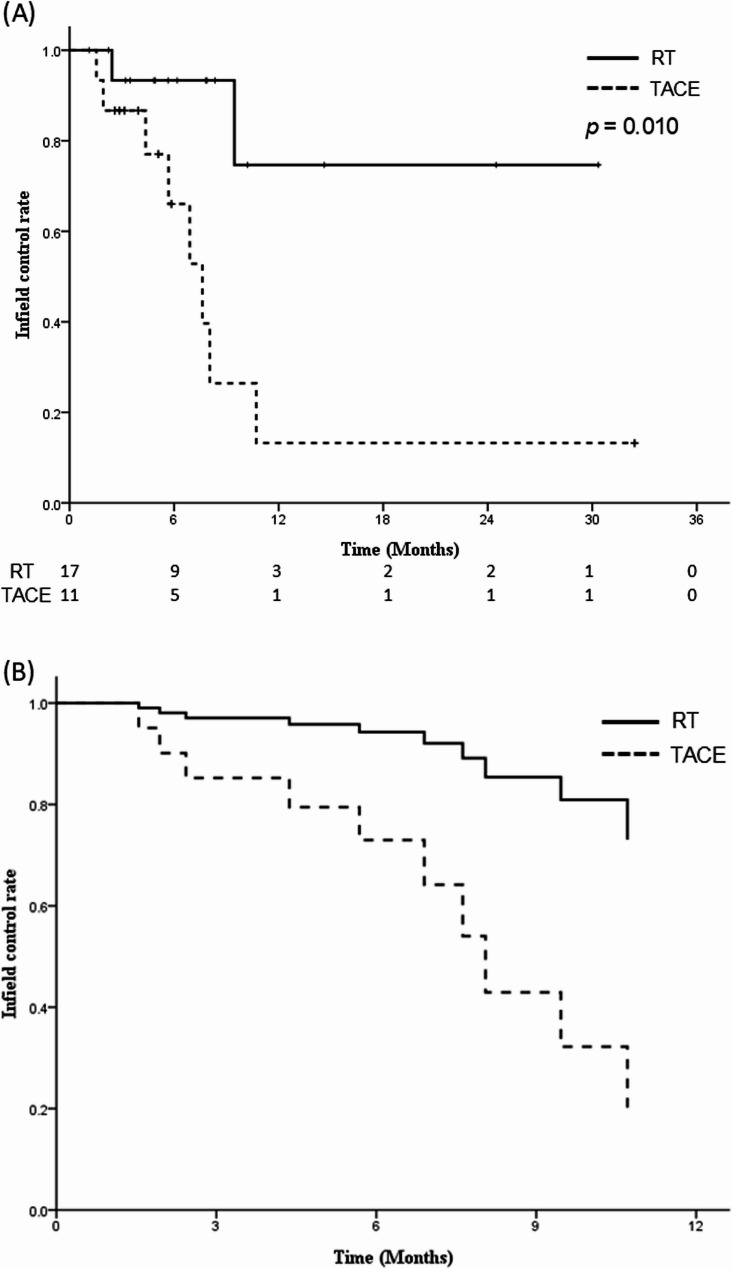
Kaplan-Meier curves of IFC **A** and multivariable cox regression of IFC **B** for patients in the RT and TACE groups

The KM curves revealed that the median IFC time was not achieved in the RT group, and the 1-year IFC rates was 74.7%, when compared to the median IFC time of 7.6 months (95% CI = 5.2–10 months) and 1-year IFC rates of 13.2% in the TACE group (Fig. 2A), with the RT group demonstrating significantly better IFC (*p* = 0.010). The multivariable analysis demonstrated that the RT group was significantly associated with better IFC outcomes (HR, 0.16; 95% CI, 0.03–0.78; *p* = 0.023) (Table 2; Supplementary Figure S3). Adjusted IFC curves for the RT and TACE groups, corrected with the confounding factors, are displayed in Fig. 2B. After applying IPTW, this advantage persisted: the IPTW-adjusted KM analysis demonstrated a significantly improved IFC in the RT group compared to the TACE group (*p* = 0.020; Supplementary Figure S4), further supporting the benefit of RT in achieving local tumor control after adjustment for baseline confounders.

Competing risk analysis demonstrated a significantly higher incidence of local failure in the TACE group compared to the RT group (Gray’s test, *p* = 0.015). At 12 months, the cumulative incidence of infield failure was approximately 61.0% for TACE group versus 14.9% for RT group (Supplementary Figure S5). In the multivariable Fine-Gray regression adjusting for tumor size (≥ 5 cm), both treatment modality and tumor size were found to be independent predictors of local failure. Patients treated with RT had a significantly lower subdistribution hazard of infield failure (subdistribution hazard ratio [sHR] 0.17; 95% confidence interval [CI]: 0.04–0.65; *p* = 0.01) compared to those receiving TACE. Similarly, tumor size ≥ 5 cm was associated with a higher risk of infield failure (sHR 3.62; 95% CI: 1.12–11.71; *p* = 0.032). These results underscore the superior local control achieved with RT, even when accounting for competing risk from death.

### Treatment sequence impact

Treatment sequence had no statistically significant impact on either OS (*p* = 0.486) or IFC (*p* = 0.720) in univariable analysis (Table 2). Subsequently, we conducted subgroup analyses to further explore potential effect modifiers based on the type of LT within each treatment sequence. In the LEN-first group, patients who received RT exhibited significantly improved IFC (median not reached) compared to those treated with TACE (median IFC: 7.6 months, *p* = 0.007). However, OS did not significantly differ between RT and TACE in this group (median OS: 8.3 vs. 14.6 months, *p* = 0.188). In contrast, within the LT-first group, no significant difference in IFC was observed between RT and TACE (median IFC: 9.5 vs. 5.7 months, *p* = 0.627). Although the median OS appeared numerically higher in the RT group (30.4 vs. 7.0 months), this difference was not statistically significant (*p* = 0.383).

### AFP kinetics

**Fig. 3:**
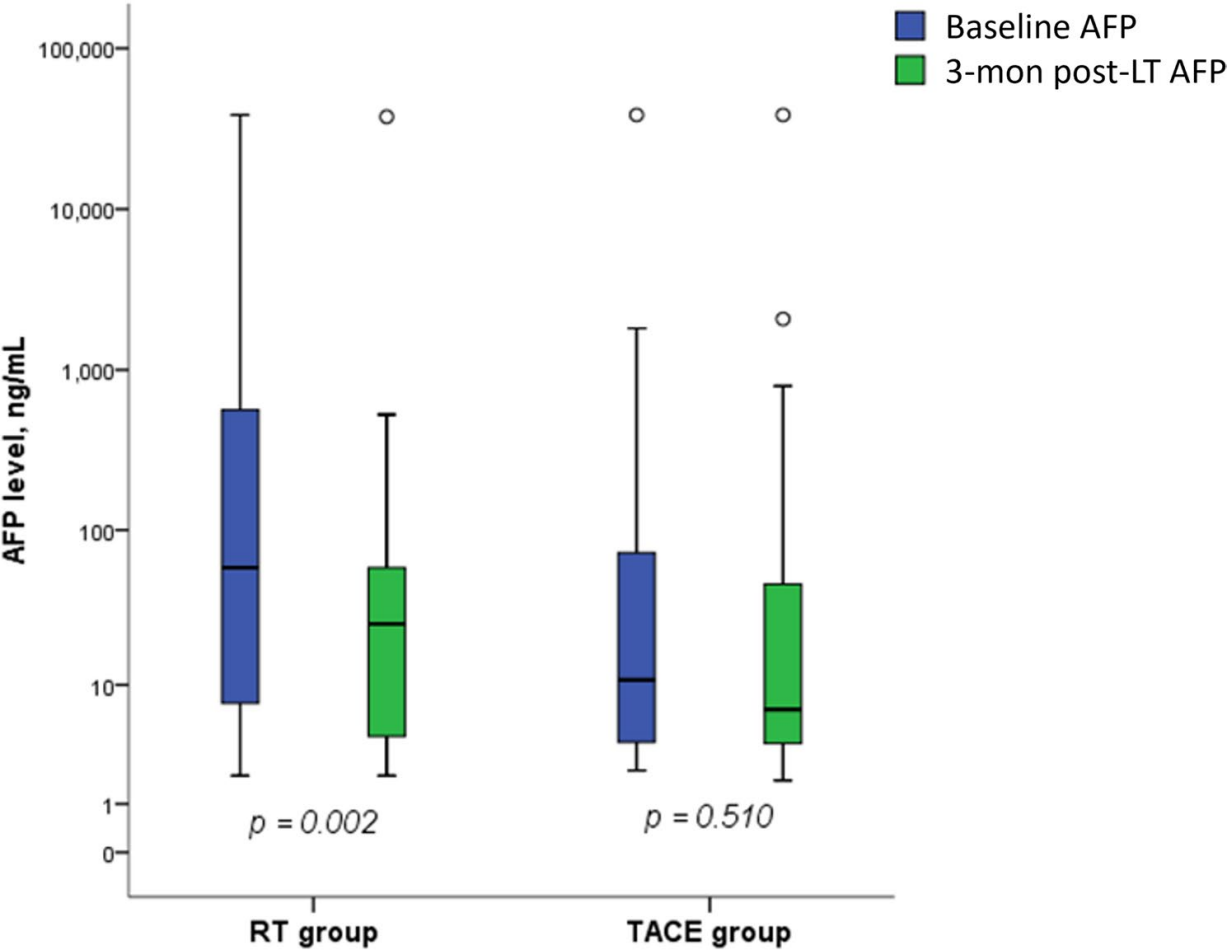
Changes in serum AFP levels at baseline and within 3 months after local therapy (referred to as “3-mon post-LT” in the figure) in the RT and TACE groups. Box plots display the median, interquartile range (IQR), and full range (whiskers), with outliers shown as open circles. AFP levels are presented on a logarithmic scale. A significant decline in AFP level was observed in the RT group (*p* = 0.002), whereas no significant change occurred in the TACE group (*p* = 0.510), suggesting a differential AFP response between treatment modalities. AFP levels are plotted with a logarithmic Y-axis. Please note that due to chart editor scaling and rendering based on original values, the visual positions of Q1 and Q3 may appear compressed or slightly inconsistent with tabulated IQR values

Serum AFP levels before and within 3 months after the completion of LT were assessed in both groups (Fig. 3). In the RT group, the median baseline AFP level was 57.8 ng/mL (interquartile range [IQR], 6.5–2941.3), which decreased to 25.3 ng/mL (IQR, 3.7–103.3) within 3 months after LT. In contrast, the TACE group showed a median basline AFP level of 10.8 ng/mL (IQR, 3.0–90.3) and a post-treatment level of 6.7 ng/mL (IQR, 3.2–50.9). The RT group demonstrated a significant decline in AFP levels following treatment (*p* = 0.002), whereas no significant change was observed in the TACE group (*p* = 0.510).

### Toxicity

Table 3 provides a Summary of the treatment related toxicity. In the RT group, one patient developed grade 3 gastrointestinal toxicity, and one patient developed grade 5 colitis, occurring 4 months post-treatment. In the TACE group, complications included two instances of grade 3 esophageal hemorrhage, one of grade 3 abdominal infection (presented as liver abscess), and one of grade 5 liver abscess. Regarding liver function tests (aspartate transaminase, alanine transaminase, albumin, or total bilirubin levels), there was no significant difference between the two groups in the incidence of grade 1–3 toxicities (52.9% vs. 46.6%; *p* = 1.00). The deterioration in the Child–Pugh score by $\:\ge\:$2 points was 5.9% in the RT group and 26.7% in the TACE group (*p* = 0.161).

**Table 3:** Treatment related toxicity according to CTCAE

	grade 1 no. (%)		grade 2 no. (%)		grade 3 no. (%)		grade 4 no. (%)		grade 5 no. (%)	
	RT	TACE	RT	TACE	RT	TACE	RT	TACE	RT	TACE
Hypoalbuminemia	-	2 (13)	5 (29)	-	1 (6)	-	-	-	-	-
Blood bilirubin increased	5 (29)	3 (20)	2 (12)	5 (33)	-	-	-	-	-	-
AST increased	6 (35)	8 (53)	-	-	1 (6)	-	-	-	-	-
ALT increased	2 (12)	3 (20)	-	-	-	-	-	-	-	-
ALK-P increased	-	-	-	-	-	-	-	-	-	-
Hypertension	2 (12)	-	2 (12)	2(13)	-	-	-	-	-	-
Abdominal infection	-	-	-	-	-	1 (7)	-	-	-	1 (7)
Gastrointestinal	2 (12)	-	1 (6)	-	1 (6)^a^	2 (13)^c^	-	-	1 (6)^d^	-
Dermatologic	3 (18)	-	1 (6)	-	1 (6)^b^	-	-	-	-	-
Fatigue	2 (12)	-	-	-	-	-	-	-	-	-

*Abbreviations*: *AST* Aspartate aminotransferase, *ALT* Alanine aminotransferase, *ALK-P* Alkaline phosphatase, *GI* Gastrointestinal, *CTCAE* Common Terminology Criteria for Adverse Events

^a^Esophageal ulcer and anal abscess

^b^Hand foot syndrome

^c^Esophageal hemorrhage

^d^Colitis

## Discussion

To the best of our knowledge, this is the first study to compare the efficacy of combining lenvatinib with either RT or TACE for treating patients with HCC. The results indicate that the RT group exhibited a significantly higher IFC rate (*p* = 0.010); however, it did not show an improvement in OS when compared to the TACE group.

Previous studies have reported 1-year OS rates for patients with HCC treated with lenvatinib and Liver-directed RT ranging from 64.1 to 68.6% [23, 24]. In contrast, the 1-year OS rate was significantly lower in the RT group (43.1%). This discrepancy can largely be attributed to the more advanced characteristics of our cohort, which included a higher ECOG performance status, higher percentage of PVTT, and extrahepatic metastases, when compared to those in other studies.

Given that our study included patients with more advanced disease, the 1-year OS rate in the TACE group was only 54.2%. In contrast, the LAUNCH trial, which assessed the efficacy of combining lenvatinib with TACE in advanced HCC, reported 1- year OS rate of 81.5% [17]. However, it is important to note that the LAUNCH trial enrolled treatment-naive patients with tumors < 10 cm, highlighting potential differences in patient demographics and tumor burden between studies.

The integration of systemic therapy with local treatments is crucial for the management of advanced HCC. Studies have consistently shown that OS is often limited by disease progression both inside and outside the liver, regardless of the local treatment (RT or TACE) [17, 23–26]. Accumulating evidence supports the synergistic effect of TACE and lenvatinib. Lenvatinib is believed to enhance the effectiveness of TACE by normalizing tumor vasculature, which in turn reduces vascular permeability and interstitial pressure and improves the delivery of embolization agents [17]. This approach highlights the potential benefits of combining systemic agents with local treatments, even in the absence of extrahepatic diseases.

Previous studies suggested that combination therapy with RT and levatinib in patients with advanced HCC had better OS, progression-free survival, and intrahepatic progression-free survival than monotherapy (RT or levatinib alone) [23, 24]. This finding may be attributed to RT’s potential to enhance the immune response against tumors. RT is increasingly recognized for its ability to induce an immune-mediated antitumor reaction, including a phenomenon known as the “abscopal effect,” where radiation treatment at one site may lead to regression at distant sites which are not directly irradiated [27]. Research has shown that RT can activate various immune responses, such as the recruitment of cytotoxic immune cells, including CD8 + T cells, NK cells, and CD8 + CD56 + natural killer T (NKT) cells, to the tumor microenvironment [28]. It has also been linked to an increased expression of IFN-γ [29] and the activation and maturation of dendritic cells, enhancing T-cell priming and potentially improving antitumor immunity [30]. Moreover, preclinical studies have shown that the antitumor efficacy of lenvatinib is enhanced by a functional immune system. Notably, the efficacy of the drug was significantly greater in immunocompetent mice when compared to those lacking immune function, with its antitumor effects diminishing when CD8 + T cells were depleted [31]. Furthermore, lenvatinib demonstrated superior antitumor activity when compared with sorafenib alone in immunocompetent mouse models. Based on these findings, it is plausible that RT indirectly enhances the efficacy of lenvatinib by boosting the immune response. Additionally, Weng et al. reported that lenvatinib enhances the efficacy of RT in HCC by inhibiting the Src/STAT3/NF-κB pathway, which mediates epithelial-mesenchymal transition and metastasis, leading to a synergistic effect [32]. This interaction suggests that RT and lenvatinib may amplify each other’s antitumor effects, partly by modulating the immune response. However, it is important to interpret these immune-related mechanisms with caution, given the retrospective nature of our study and the absence of direct immune profiling or biomarker data. As such, these proposed interactions should be regarded as hypothesis-generating rather than definitive.

In our study, the RT group demonstrated superior outcomes compared to the TACE group in terms of IFC rate. Our finding may cautiously suggest that when lenvatinib is initiated before LT, RT may provide superior local control compared to TACE. Preclinical studies have demonstrated that vascular endothelial growth factor (VEGF) inhibition can transiently normalize tumor vasculature, thereby improving perfusion, enhancing oxygenation, and reducing interstitial fluid pressure—all of which can sensitize tumors to RT. This phenomenon, known as vascular normalization, has been well-characterized in anti-VEGF strategies [33, 34]. Lenvatinib, which targets VEGF receptors (VEGFR1-3) among other kinases, has been shown to promote vascular remodeling and improve the intratumoral microenvironment in HCC models [35]. These changes may reduce tumor hypoxia and enhance oxygen-dependent DNA damage, thereby contributing to the synergistic effects observed when lenvatinib is combined with RT. In TACE-based protocols, lenvatinib preloading has been proposed to improve intraarterial drug distribution, and suppress the VEGF surge caused by post-embolization hypoxia [36, 37]. However, the optimal timing remains unclear. Prolonged lenvatinib administration may over-prune tumor vessels, impair drug delivery, and reduce TACE efficacy [38]. These complexities highlight key differences in how lenvatinib interacts with RT versus TACE. Notably, unlike TACE, RT does not rely on intraarterial delivery, and may therefore benefit more consistently from improved perfusion. In addition to radiographic outcomes, we observed a differential biomarker response between treatment groups. The RT group exhibited a more favorable AFP kinetic profile after LT compared to the TACE group. This difference may reflect distinct biological effects associated with each modality. The AFP reduction seen in the RT group supports a potential mechanistic link between lenvatinib-induced radiosensitization and enhanced intrahepatic tumor control. These mechanistic and biomarker-based findings collectively support the observed local control advantage with RT and warrant further investigation in prospective studies. For OS, the RT group did not demonstrate improved outcomes, possibly because it included a higher proportion of patients with advanced BCLC stages and more pronounced PVTT.

There is a discrepancy between the Cox model and the Fine–Gray competing risk model, particularly regarding tumor size, which was statistically significant only in the Fine–Gray model but not in the Cox model. This difference may reflect the different assumptions and endpoints inherent to each method. The Cox model does not account for death as a competing event, whereas the Fine–Gray model explicitly incorporates it into the analysis. Given that a significant proportion of patients may die before experiencing local failure, the Fine–Gray model may offer a more accurate representation of IFC dynamics in this population. Taken together, these findings suggest that treatment modality consistently impacts IFC, regardless of statistical approach. Tumor size, while not significant in the Cox model, emerged as an independent predictor in the Fine–Gray model, supporting its potential relevance to local failure risk, particularly when accounting for competing mortality risks.

Unexpectedly, patients with ALBI grade ≥ 2 showed better OS than those with ALBI grade 1, a finding that contradicts established prognostic models. To explore this paradox, we performed several supplementary analyses (Supplementary Table S2). When treated as a continuous variable, ALBI score was not significantly associated with OS. Excluding patients with early non–liver-related events, including liver transplantation and early loss to follow-up, did not alter the observed trend. Further, alternative stratifications using Child–Pugh class or modified ALBI grade demonstrated outcomes consistent with better survival among patients with preserved liver function (Supplementary Figure S6). Notably, a higher proportion of ALBI ≥ 2 patients were in the RT group, which exhibited numerically worse OS than the TACE group. This reduces the likelihood that favorable treatment allocation explains the paradoxical trend. Taken together, these results suggest that the apparent survival benefit in the ALBI ≥ 2 group likely reflects threshold-related artifacts or small-sample variation rather than a true biological association.

While most patients in our study did not experience severe adverse events, two grade 5 adverse events were noted: colitis and Liver abscess. Notably, the SELECT and REFLECT trials reported that fistula or gastrointestinal perforation occurs in approximately 2% of patients treated with lenvatinib [11]. On November 22, 2021, the Pharmacovigilance Risk Assessment Committee of the European Medicines Agency recommended that colitis should be listed as an uncommon adverse event in lenvatinib’s package leaflet [39]. In our study, one patient in the RT group developed ascending colon colitis 4 months after RT, which subsequently led to bowel perforation. Despite undergoing loop ileostomy and drainage, the patient died. A review of this patient’s RT plan confirmed that normal organ constraints were within safe limits (V30 Gy = 0.03 cc), Suggesting that stricter normal organ dose constraints should be followed during the concurrent use of RT and lenvatinib, especially in the gastrointestinal tract. Additionally, Liver abscess formation has been reported in 1.3 to 2.4% of patients in previous TACE studies [40]. The incidence of Liver abscess was higher in our cohort, developing in 2 of 11 (18.2%) patients. Notably, one patient in our TACE group, who had previously undergone a liver transplant, Succumbed to recurrent Liver abscesses 5 months post-TACE procedure, highlighting a potentially increased risk in certain patient populations. The LAUNCH trial suggested that TACE combined with lenvatinib may increase liver toxicity [17]. However, previous studies have not observed a similar increase in liver toxicity with RT combined with lenvatinib [23, 24]. Liver function may be better preserved in the RT group when compared to the TACE group, as indicated by the lower proportion of patients experiencing a ≥ 2-point deterioration in the Child–Pugh score. However, this difference was not statistically significant, likely due to the limited sample size.

The present study had a few limitations. First, its retrospective nature and relatively small sample size may have impacted the generalizability of the findings. Second, there was variability in the dose and fractionation of RT. The Current National Comprehensive Cancer Network guidelines offer a range of doses and prescribing principles rather than specific dosages, allowing clinicians’ flexibility based on individual patient tolerance, especially for reserved liver function and volume. In our study, we administered the maximum tolerable dose for HCC treatment. Lastly, we lacked imaging-based biomarkers of tumor response such as vascularity or necrosis rates, which could have offered additional mechanistic insight.

## Conclusions

Our study suggests that combining RT with lenvatinib improves IFC and potentially offer a trend toward better liver function preservation compared to TACE plus lenvatinib in patients with advanced HCC. These findings serve as a pilot investigation indicating the potential synergistic anti-tumor efficacy of RT and lenvatinib in advanced HCC. However, further prospective randomized trials are warranted to validate these results and provide more robust evidence in this cohort.

## Supplementary Information

Supplementary Material 1.

## Data Availability

The data from this study can be obtained upon request from the corresponding author in an anonymized format following a data privacy review. Due to privacy regulations, the data are not publicly accessible.

## Abbreviations

ABC: Active breathing control
AFP: Alpha-fetoprotein
CFRT: Conventional fractionated radiotherapy
CT: Computed tomography
CTCAE: Common terminology criteria for adverse events
CTV: Clinical target volume
ECOG: Eastern cooperative oncology group
Gy: Gray
GTV: Gross tumor volume
HCC: Hepatocellular carcinoma
IFC: Infield control
IPTW: Inverse-probability-of-treatment weighting
KM: Kaplan–Meier
LEN: Lenvatinib
LT: Local therapy
mRECIST: Modified response evaluation criteria for solid tumors
MRI: Magnetic resonance imaging
NAFLD: Non-alcoholic fatty liver disease
NKT: Natural killer T
OS: Overall survival
PTV: Planning target volume
PVTT: Portal vein tumor thrombosis
RT: Radiotherapy
SBRT: Stereotactic body radiation therapy
TACE: Transarterial chemoembolization
VEGF: Vascular endothelial growth factor
