# Supplementary material for: Lenvatinib in combination with radiotherapy versus lenvatinib with transarterial chemoembolization for advanced hepatocellular carcinoma
Source: BMC Cancer. 2025 Sep 30;25:1449. doi: 10.1186/s12885-025-14931-1 (PMC12482564; doi:10.1186/s12885-025-14931-1)

Supplementary Table S1. Baseline covariate balance before and after inverse probability of treatment weighting.

Covariate	Unadjusted SMD	Adjusted SMD
PVTT	0.322	0.0398
EM	0.102	0.0537
Etiology	0.666	0.3511
BCLC stage	0.298	0.0397
Recurrent disease	0.216	0.0482

Abbreviations: BCLC, Barcelona Clinic Liver Cancer; EM, extrahepatic metastasis; PVTT, portal vein tumor thrombosis; SMD, standardized mean difference

Supplementary Table S2. Univariable analysis of OS based on liver function scales

	Full cohort		Cohort (Excl. ENLRE)	
Variable	HR (95% CI)	*p*	HR (95% CI)	*p*
ALBI grade (≥2 vs. 1)	0.43 (0.15-1.17)	0.099	0.36 (0.13-0.996)	0.049
ALBI score (continuous)	0.93 (0.40-2.13)	0.857	0.85 (0.36-2.01)	0.717
mAIBI grade (≥2b vs. 1–2a)	1.96 (0.77-5.00)	0.160	1.85 (0.72-4.73)	0.200
Child–Pugh class (B vs. A)	2.03 (0.75-5.52)	0.165	1.93 (0.71-5.25)	0.198

Abbreviations: OS, overall survival; ENLRE = Early non–liver-related events, including liver transplantation and early loss to follow-up; ALBI, albumin-bilirubin; mALBI, modified albumin-bilirubin grade (ALBI grade 2 subdivided into 2a [–2.60 to –2.27] and 2b [> –2.27] based on standard cutoffs); HR, hazard ratio; CI, confidence interval.

Supplementary Figure S1. Forest plot of multivariable Cox regression analysis showing hazard ratios and 95% confidence intervals for predictors of overall survival. The x-axis is on a logarithmic scale, and the dashed vertical line at HR = 1 represents the reference point of no effect.

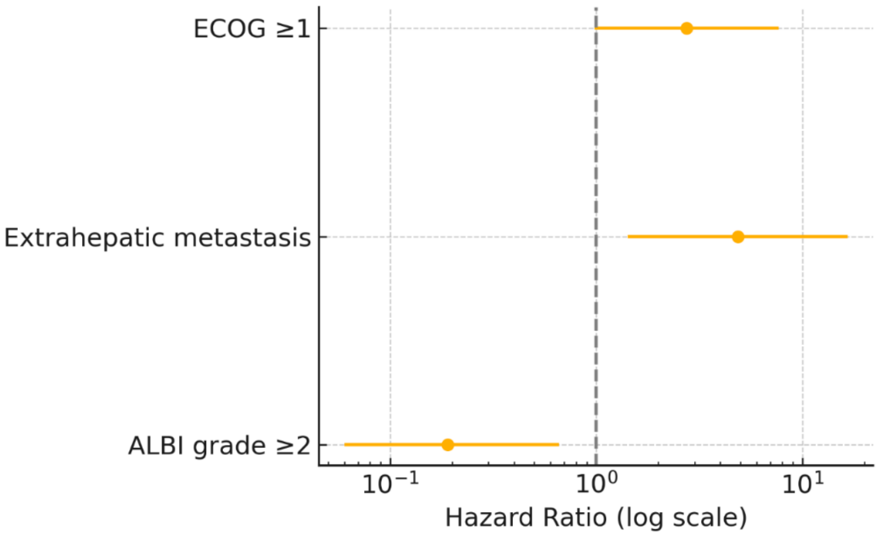

Supplementary Figure S2. Weighted Kaplan–Meier curves for OS in patients treated with RT versus TACE, following IPTW adjustment. No significant difference in OS was observed between the two groups (p = 1.000). The number of patients at risk at each time point is shown below the graph.

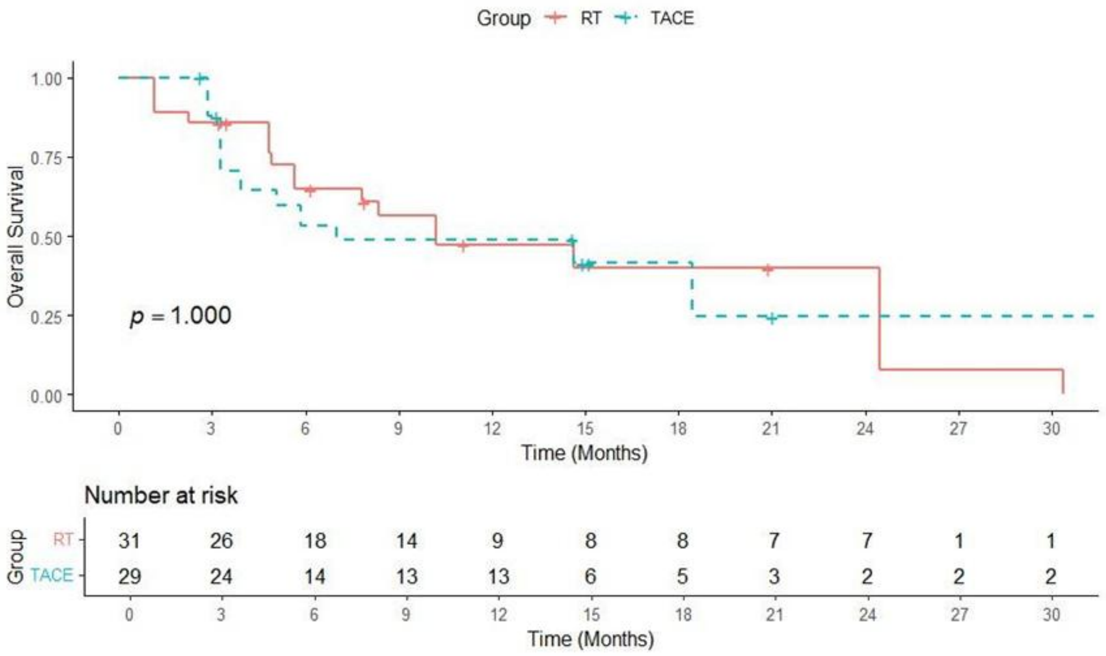

Supplementary Figure S3. Forest plot of multivariable Cox regression analysis showing hazard ratios and 95% confidence intervals for infield control. RT was associated with a significantly lower hazard of infield failure compared to TACE. The x-axis is on a logarithmic scale, and the dashed vertical line at HR = 1 represents the reference point of no effect.

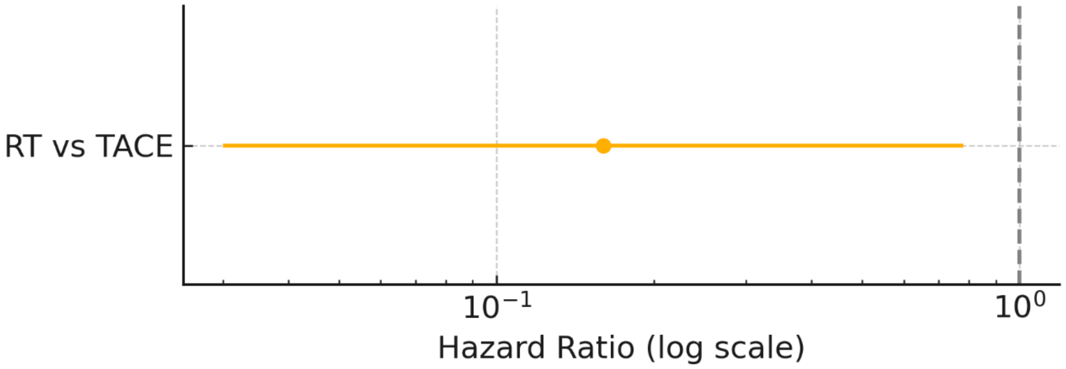

Supplementary Figure S4. Weighted Kaplan–Meier curves for IFC in patients receiving RT versus TACE after IPTW adjustment. The RT group showed significantly better IFC compared to the TACE group (***p*** = 0.020). The number at risk at each time point is shown below the graph.

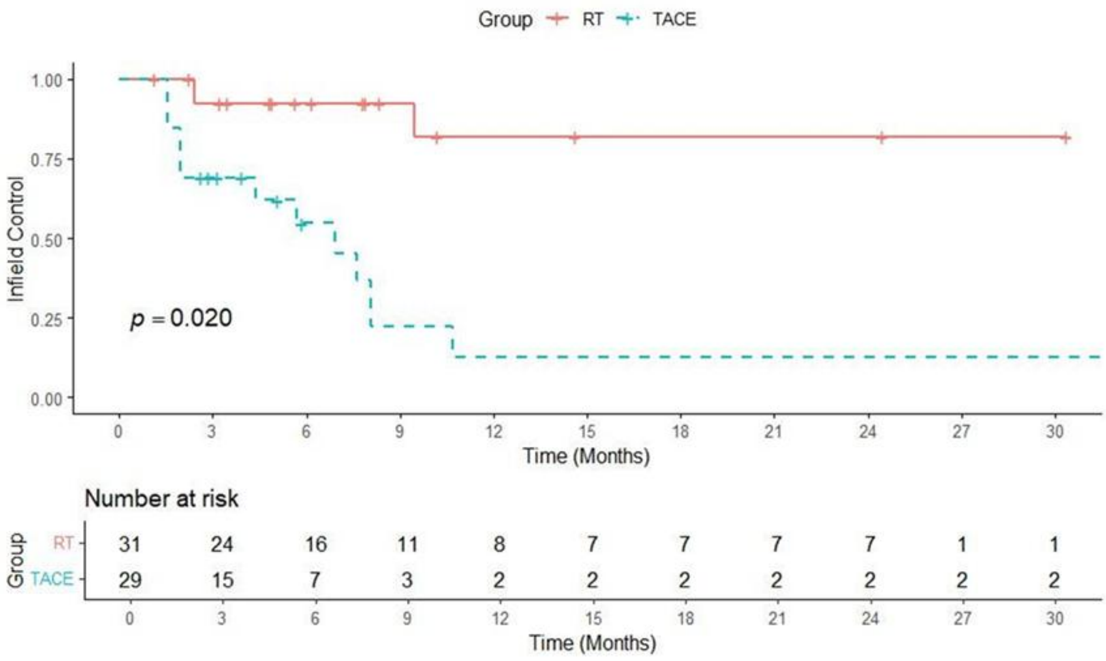

Supplementary Figure S5. Cumulative incidence of infield failure in patients treated with RT versus TACE. The TACE group showed a significantly higher cumulative incidence of local failure compared to the RT group ($p = 0.015$), suggesting superior local control with RT over time.

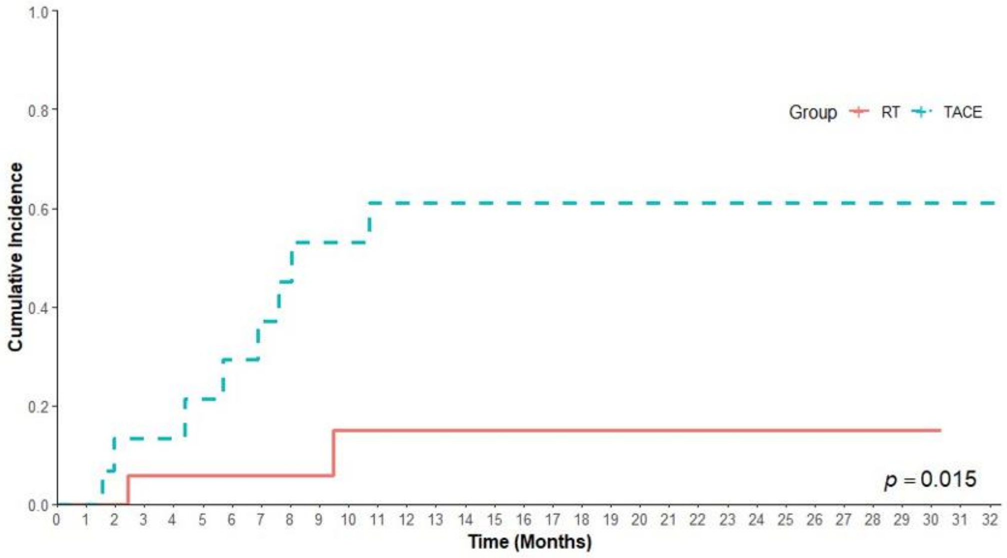

RT	17	17	16	14	12	10	9	8	6	5	4	3	3	3	3	2	2	2	2	2	2	2	2	2	1	1	1	1	1	1		
TACE	15	15	13	11	9	8	5	4	3	2	2	1	1	1	1	1	1	1	1	1	1	1	1	1	1	1	1	1	1	1	1	1

Supplementary Figure S6. Kaplan–Meier curves of overall survival stratified by liver function scales. (A) ALBI grade; (B) modified ALBI (mALBI) grade; (C) Child–Pugh class.

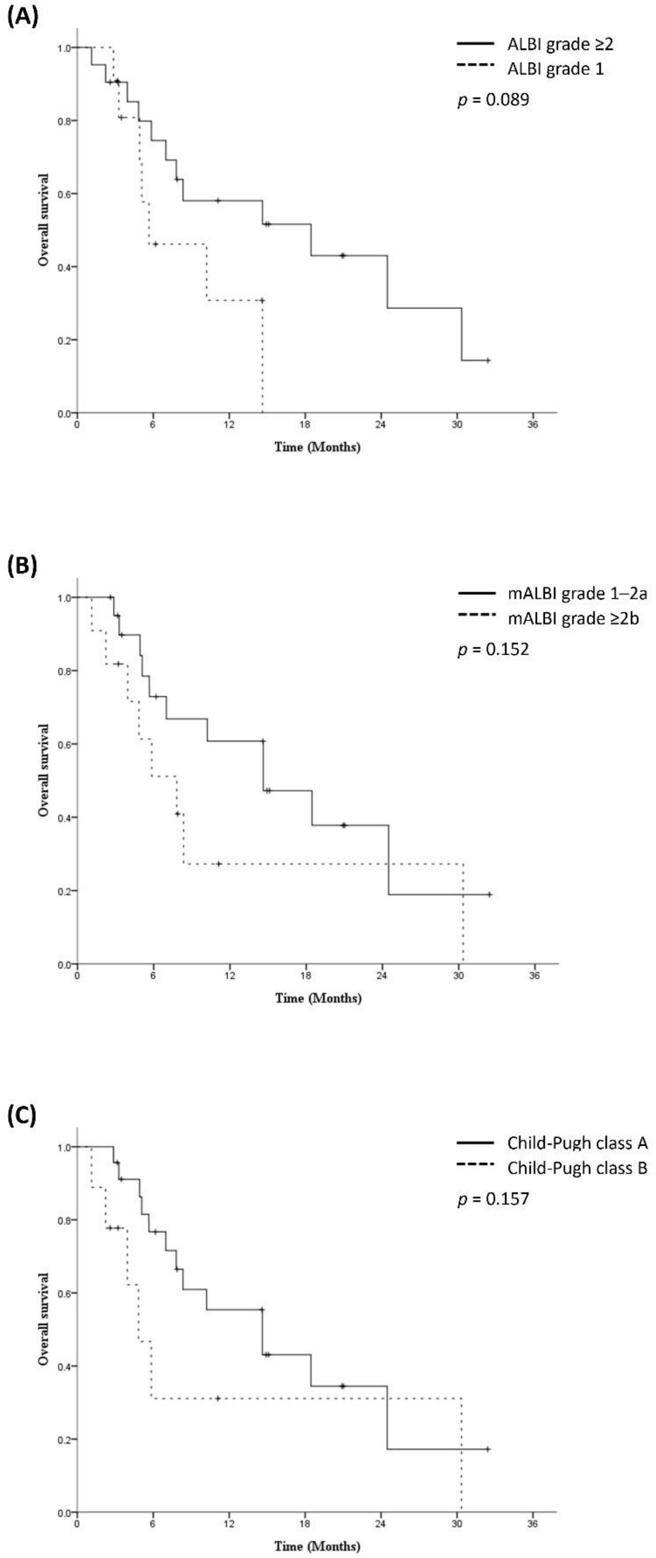

Supplement: Supplementary file 1 — Supplementary Material 1. [file 12885_2025_14931_MOESM1_ESM.pdf]
